# Editorial: Cold Pressed Oils: A Green Source of Specialty Oils

**DOI:** 10.3389/fnut.2021.836651

**Published:** 2022-02-11

**Authors:** Alessandra Durazzo, Mohamed Fawzy Ramadan, Massimo Lucarini

**Affiliations:** ^1^CREA-Research Centre for Food and Nutrition, Rome, Italy; ^2^Umm Al-Qura University, Makkah, Saudi Arabia; ^3^Zagazig University, Zagazig, Egypt

**Keywords:** nutraceuticals, healthy oils, specialty oils, cold technologies, nutrients, adulteration, functional food, food composition databases

The need for widely usable bioactive lipids and natural antioxidants continues to grow. Methods used for oil extraction may alter minor constituents that have functional properties and contribute to oil stability. In recent years, consumer demand for cold pressed oils as an alternative to conventional products has increased. This is probably due to the perception of these oils as natural, nutritional, and safe food products with better nutritive and healthy properties.

Cold pressed oils are easy to obtain. The process does not involve heat treatment or solvent extraction. Therefore, the cold pressing technique results in a product that is free of organic solvents. Mechanical pressing does not require much energy and meets well the needs of small- and medium-sized companies. Cold pressed oils are promoted as specialty oils and are usually available on the market at a higher price. Cold pressed oil products are reported to contain pro- and anti-oxidative compounds.

In recent years, consumption of cold pressed oils has been increasing. The international market already includes numerous cold pressed oils (e.g., grape seed, virgin olive, rapeseed oil, soybean, paprika, sesame, black cumin seed, amaranth, orange, lemon seed, grapefruit, pomegranate, chia seed, corn, sunflower, safflower, pumpkin, hazelnut, pistachio, walnut, pecan, clove, berries, oregano, rosehip, carrot, coriander, peanut, niger, rice bran, avocado, tomato seed, and argan). However, due to high prices of cold pressed oils, they are exposed to adulteration with cheaper and lower-quality refined oils of vegetal origin. Several strategies have been applied to monitor and control the authenticity of cold pressed oils for reasons of both human health and demand, as well as concern for controlling product quality in industrial laboratories. Among these, new frontiers are open toward the use of rapid analytical methods and chemometrics.

This Research Topic aims to report on recent advances in cold pressed oils as specialty oils, mainly focusing on their composition, physicochemical characteristics, organoleptic attributes, nutritional quality, oxidative stability, food applications, and functional and health-promoting traits.

The main explored topics are as follows: (i) evaluation procedures and quality attributes (e.g., distinction between virgin and extra virgin), yield, quality parameter, and physicochemical properties, sensory and color attributes; (ii) compounds of nutritional and nutraceutical characters in cold pressed oils; (iii) oxidative stability; (iv) impact of cold technologies: extracting and processing parameters; (v) storage and shelf life evaluation; (vi) adulteration and authenticity; (vii) cold pressed cake as functional and value-added product; (viii) consumer perception; (ix) health benefits; (x) safety aspects and potential adverse reaction evaluations; (xi) conventional and innovative fields of applications; (xii) food composition database and dedicated databases; (xiii) regulatory challenges; and (xiv) current and future applications and research needs.

Fratianni et al. described the fatty acid composition, antioxidant, and *in vitro* anti-inflammatory activity of five cold pressed *Prunus* seed oils and their anti-biofilm effect on pathogenic bacteria. Another example is given by Wang et al. who compared the antioxidant and antibacterial activities of camellia oil from Hainan with camellia oil from Guangxi, olive oil, and peanut oil. Gharby and Charrouf reviewed the chemical composition, extraction process, and quality control of argan oil.

It is worth mentioning the study of Tsao et al. on the application of OXITEST for the prediction of shelf-lives of selected cold pressed oils. Results first revealed that the UFA (Unsaturated Fatty Acid) portion was partially correlated with the shelf-lives of selected expeller-pressed seed oils as estimated by the OXITEST. Benincasa et al. reported the identification of phenolic compounds in spray-dried olive mill wastewater; the study showed that the use of dehumidified air as a drying medium, with the addition of maltodextrin, results an effective way to obtain a phenol-rich powder.

## Author Contributions

All the authors listed made a substantial, direct, and intellectual contribution to the article and approved it for publication.

## Conflict of Interest

The authors declare that the research was conducted in the absence of any commercial or financial relationships that could be construed as a potential conflict of interest.

## Publisher's Note

All claims expressed in this article are solely those of the authors and do not necessarily represent those of their affiliated organizations, or those of the publisher, the editors and the reviewers. Any product that may be evaluated in this article, or claim that may be made by its manufacturer, is not guaranteed or endorsed by the publisher.

